# Cost-Effectiveness of Sugammadex Versus Neostigmine to Reverse Neuromuscular Blockade in a University Hospital in Taiwan: A Propensity Score-Matched Analysis

**DOI:** 10.3390/healthcare11020240

**Published:** 2023-01-12

**Authors:** Winnie Lan, Ka-Wai Tam, Jui-Tai Chen, Juan P. Cata, Yih-Giun Cherng, Yun-Yun Chou, Li-Nien Chien, Chia-Li Chang, Ying-Hsuan Tai, Lu-Min Chu

**Affiliations:** 1 Department of Anesthesiology, Mackay Memorial Hospital, Taipei 104, Taiwan; 2 Department of General Medicine, Shuang Ho Hospital, Taipei Medical University, New Taipei City 235, Taiwan; 3 Shared Decision Making Resource Center, Shuang Ho Hospital, Taipei Medical University, New Taipei City 235, Taiwan; 4 Cochrane Taiwan, Taipei Medical University, Taipei 110, Taiwan; 5 Division of General Surgery, Department of Surgery, Shuang Ho Hospital, Taipei Medical University, New Taipei City 235, Taiwan; 6 Division of General Surgery, Department of Surgery, School of Medicine, College of Medicine, Taipei Medical University, Taipei 110, Taiwan; 7 Department of Anesthesiology, Shuang Ho Hospital, Taipei Medical University, New Taipei City 235, Taiwan; 8 Department of Anesthesiology, School of Medicine, College of Medicine, Taipei Medical University, Taipei 110, Taiwan; 9 Department of Anesthesiology and Perioperative Medicine, The University of Texas MD Anderson Cancer Center, 1515 Holcombe Blvd, Unit 409, Houston, TX 77030, USA; 10 Institute of Health and Welfare Policy, College of Medicine, National Yang Ming Chiao Tung University, Taipei 112, Taiwan; 11 Graduate Institute of Data Science, College of Management, Taipei Medical University, Taipei 110, Taiwan; 12 Health Data Analytics and Statistics Center, Office of Data Science, Taipei Medical University, Taipei 110, Taiwan

**Keywords:** decurarization, economic analysis, general anesthesia, perioperative efficiency, turnover rate

## Abstract

Sugammadex has several pharmacological advantages over neostigmine, including faster reversal of neuromuscular blockade and fewer adverse effects. However, the economic impact of sugammadex remains controversial due to the considerable heterogeneity of study designs and clinical settings in previous studies. In a post-hoc analysis of a randomized controlled trial, we evaluated patients who underwent elective surgeries and general anesthesia with endotracheal intubation in a medical center in Taiwan between March 2020 and August 2020. Patients were divided into either the sugammadex or neostigmine group based on the neuromuscular blocking drug used. Propensity score matching was used to balance the baseline patient characteristics between the two groups. The patient’s recovery from anesthesia and the putative cost-effectiveness of sugammadex versus neostigmine was assessed. Derived cost-effectiveness using personnel costs in the operating room and the post-anesthesia care unit was estimated using multiple linear regression models. A total of 2587 and 1784 patients were included before and after matching, respectively. Time to endotracheal extubation was significantly shorter in the sugammadex group (mean 6.0 ± standard deviation 5.3 min) compared with the neostigmine group (6.6 ± 6.3 min; *p* = 0.0032). In addition, the incidence of bradycardia was significantly lower in the sugammadex group (10.2%) compared with the neostigmine group (16.9%; *p* < 0.001). However, the total costs were significantly lower in the neostigmine group (50.6 ± 21.4 United States dollars) compared with the sugammadex group (212.0 ± 49.5 United States dollars). Despite improving postoperative recovery, the benefits of sugammadex did not outweigh its higher costs compared with neostigmine, possibly due to the low costs of labor in Taiwan’s healthcare system.

## 1. Introduction

The European Union first approved the use of sugammadex for reversing neuromuscular blockade in 2008. This modified γ-cyclodextrin encapsulates free molecules of certain types of muscle relaxants, forming stable complexes and, thus, more rapidly reversing neuromuscular blockade compared with traditional reversal agents, such as neostigmine [1,2].

The clinical benefits of sugammadex versus neostigmine have been evaluated across a range of features, including rates of postoperative pulmonary complications, residual paralysis, postoperative nausea and vomiting (PONV), 30-day readmission, and cognitive function [3,4,5,6,7,8,9,10,11,12,13,14,15,16]. A series of cost-effectiveness analyses in various healthcare settings were also performed to determine whether it was more economical for patients and healthcare systems to use sugammadex in routine clinical practice or for certain patient populations [6,17,18,19,20,21,22,23]. However, due to the methodological diversity of study designs and variations in healthcare systems, studies have delivered conflicting outcomes, and the use of sugammadex is now somewhat controversial. Hence, the economic benefits of sugammadex have not been fully evaluated due to some limitations in previous studies, including small sample size (*n* < 1000) [6,17,18,19,20] and restriction to specific types of surgery [17,21]. In addition, most previous studies analyzed the cost-effectiveness of sugammadex in the hospitals of western countries (two in Italy [6,17] and three in the United States [20,22,23]). The economic impact of sugammadex remains unclear in Asian healthcare systems due to limited published data [21].

In this study, we sought to clarify and investigate the potential cost-effectiveness of sugammadex versus neostigmine using real-world data in Taiwan. We reasoned that the use of sugammadex could accelerate the patient’s emergence from general anesthesia, increasing operating turnover rates and, therefore, reducing total medical costs compared to neostigmine. Based on current evidence [6,17,18,19,20,21,22,23], we hypothesized that the administration of sugammadex was associated with a shorter time to recovery and lower total costs related to surgical patients undergoing general anesthesia.

## 2. Materials and Methods

### 2.1. Data Source

This study was a retrospective sequential analysis of a published randomized controlled trial registered on Clinicaltrials.gov (identifier: NCT04272177) [24]. The two-center randomized controlled trial estimated the potential effect of patient decision aids (PtDA) on the choice of neuromuscular blocking reversal agents. Eligible patients were randomly allocated into the classical explanation group or the PtDA-assisted explanation group before deliberation in pre-anesthesia assessments. Participants were then allowed to choose either neostigmine or sugammadex after their consultation. According to the hospital policy, sugammadex would be introduced to patients as a self-paid reversal agent during pre-anesthesia consultations. In the randomized controlled trial, the anesthesiologist and nurse anesthetist who administered the neuromuscular blocking and reversal agents were blinded to the decision-making process. In addition, the post-anesthesia care unit (PACU) nurse was also masked to the group assignment.

### 2.2. Study Design and Participants

**Figure 1:**
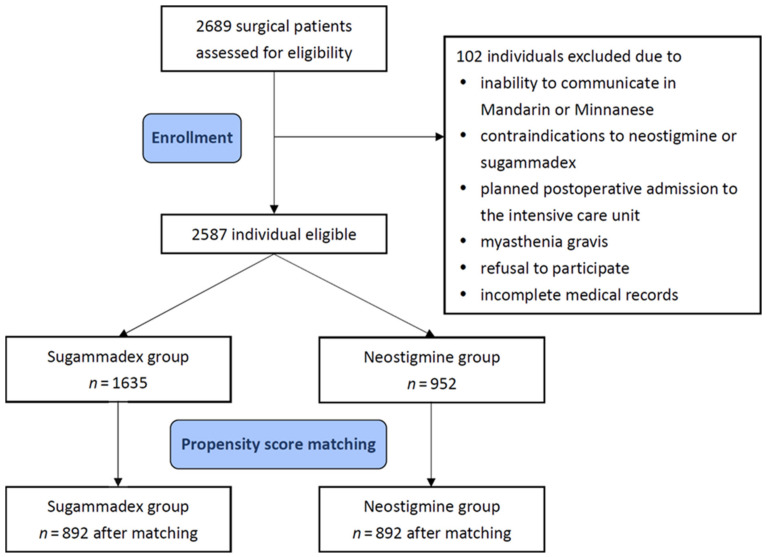
Flow diagram for patient inclusion.

The current study was approved by the Institutional Review Board of Taipei Medical University (approval no. TMU-JIRB-N201909073; date of approval on 24 October 2019). The Institutional Review Board waived written informed consent due to the retrospective nature of this research. All methods were performed following the Declaration of Helsinki 2013. Inclusion criteria were (1) patients who were admitted for elective surgeries requiring general anesthesia with endotracheal intubation at Shuang Ho Hospital, Taipei Medical University, from 1 March 2020 to 31 August 2020, (2) ≥20 years old, and (3) participated in pre-anesthesia assessments. Patients were excluded for the following reasons: inability to communicate in Mandarin or Minnanese, contraindications to neostigmine or sugammadex (known drug allergy, estimated glomerular filtration rate <30 mL·min·1.73 m^−2^, and aspartate aminotransferase or alanine aminotransferase >2 times upper limits of normal), planned postoperative admission to the intensive care unit (ICU) for mechanical ventilation, myasthenia gravis, refusal to participate, and incomplete medical records (Figure 1). Patients were classified into either the sugammadex group or the neostigmine group based on the reversal agent used.

### 2.3. Anesthesia Management Protocol

All patients received propofol 1–2 mg·kg^−1^ and fentanyl 1–3 μg·kg^−1^ for induction of general anesthesia. Neuromuscular blockade was achieved using rocuronium 0.6–1 mg·kg^−1^ in the sugammadex group, and either rocuronium 0.6–1 mg·kg^−1^ or cisatracurium 0.1–0.2 mg·kg^−1^ in the neostigmine group. General anesthesia was maintained using inhalational sevoflurane or desflurane, with the concentration adjusted based on the patient’s vital signs and clinical judgment. Sugammadex 2 mg·kg^−1^ or neostigmine 0.05 mg·kg^−1^/glycopyrrolate 0.01 mg·kg^−1^ were given to reverse neuromuscular blockade after the train-of-four count recovered to ≥2. Extubation was attempted once the train-of-four ratio was over 0.9 or patients obeyed verbal commands (sustained handgrip or head lift for 5 s). After surgery, a modified Aldrete score >9 was required for discharge from the PACU.

### 2.4. Covariates for Model Adjustment

The electronic medical database was used to evaluate patient and clinical characteristics, including age, sex, body mass index (BMI), and serum creatinine levels. We also collected data on types of surgery performed, intravenous and inhalational medications used, duration of surgery and anesthesia, length of PACU stay, postoperative adverse events, and oxygenation supply techniques at the PACU based on the electronic anesthesia and PACU records. This data was extracted by an independent physician (W.L.), who was not involved in the data analysis. The quality of the datasets was validated using random samples by other authors.

### 2.5. Outcome Assessment

The primary outcome was time to extubation, which was defined as the interval from the administration of neuromuscular blockade reversal agents to endotracheal tube removal. The secondary outcomes were the duration of surgery and anesthesia, length of PACU stay, and adverse events in the PACU. Adverse events included PONV, bradycardia (a resting heart rate of <50 beats per minute), oxygen desaturation (peripheral oxygen saturation < 93%), use of a nasal cannula, a simple mask, or continuous positive airway pressure therapy for supplemental oxygen, and the need for tracheal re-intubation. In the PACU, the data of heart rate and peripheral oxygen saturation were recorded in 5-min intervals. In the original randomized trial, the adverse events were assessed by a certificated nurse anesthetist blinded to the choice of a reversal agent. 

### 2.6. Cost-Effectiveness Analysis

It was thought that the reduced amount of time spent in the operating room (OR), as caused by sugammadex, could create cost savings for our healthcare system and patients. As detailed by prior studies [12,18,20], per-minute operating room costs were calculated using the combined labor cost when a consultant surgeon, a surgical resident, a consultant anesthetist, a scrub nurse, a circulating nurse, and a nurse anesthetist were present. One registered nurse was considered present during the regular setting of the PACU. The average monthly salary of each position was estimated by our human resource office in 2022, then calculated on a per-minute basis and converted to United States dollars (USD) using the average exchange rate between January and March 2022 (1 USD = 27.83 New Taiwan dollars).

The Department of Pharmacy in our hospital provided information regarding the unit price of neuromuscular blocking and reversal agents, which were used for general anesthesia on a self-paid basis. The total costs of each drug were calculated based on the dose used for each individual. Economic benefits were defined as the difference in personnel costs derived from the mean difference in time to endotracheal extubation between the two groups. The associated costs of medication-related adverse events (e.g., anaphylaxis, bradycardia, pulmonary complications, readmission, or intensive care) were not included in our analyses due to the relatively low incidence rate, considerable interindividual variation in relevant medications and interventions and insufficient evidence in estimating the potential costs.

### 2.7. Statistical Analysis

Based on a previous study, at least 84 patients are needed in each group to detect a mean difference of 2.7 min in time to extubation between the sugammadex and neostigmine groups, accepting a type I error of 5% and a type II error of 10%, with an anticipated mean time to extubation of 9.0 ± standard deviation (SD) 5.4 min in the neostigmine group [25,26]. It should be noted that the number of subjects enrolled in the matched cohort substantially exceeded the minimal sample size needed. Continuous variables were expressed as the mean ± SD, while categorical variables were reported as numbers and percentages. Patients in the sugammadex group were matched to patients in the neostigmine group in a 1:1 ratio using the nearest neighbor matching algorithm within a tolerance limit of 0.05 and without replacement to balance the distributions of age, sex, BMI, and rocuronium dose between the two groups. Baseline patient characteristics were compared between groups using the absolute standardized mean difference (ASMD) [27]. Imbalance was defined as an ASMD value greater than 0.2. Multivariable linear regression models were used to estimate the intergroup differences using the GENMOD procedure of SAS software, version 9.4 (SAS Institute Inc., Cary, NC, USA) in time to extubation, duration of surgery and anesthesia, time to leave the operating room, length of PACU stay, derived personnel costs, and drug costs. The normality of included variables was checked by Kolmogorov-Smirnov tests and Shapiro-Wilk tests. Non-normally distributed variables were log-transformed in the multivariable models to reduce the distribution skewness, including age, body mass index, and rocuronium dose. A two-sided *p*-value < 0.05 was considered statistically significant. A Bonferroni correction to the significance criterion was applied for multiple testing adjustments.

## 3. Results

### 3.1. Baseline Patient Characteristics

A total of 2689 consecutive patients were screened for eligibility, and 2587 were selected for propensity score matching (Figure 1). Before matching, patients in the sugammadex group were more likely to be older, female, have a higher BMI, and have a lower serum creatinine level than their counterparts (Table 1). In addition, patients using sugammadex had higher proportions of general and neurological surgeries and lower proportions of otolaryngological, gynecological, and urological surgeries. Patients in the sugammadex group also received greater doses of rocuronium during surgery. The matching procedure generated 892 matched pairs for analyses. It should be noted that all of the baseline patient and clinical characteristics were balanced after matching. A total of 39 (4.1%) patients in the neostigmine group received cisatracurium as the neuromuscular blocking agents in the original cohort and were excluded after the propensity score matching analysis. Table 2 and Supplementary Table S1 show the drug costs and personnel costs which were used for the cost-effectiveness analyses. In the matched cohort, the mean dosage of sugammadex used in the sugammadex group was 143.9 ± SD 0.1 mg, while the mean dosage of neostigmine in the neostigmine group was 2.6 ± SD 0.3 mg.

### 3.2. Operating Room Turnover Time

In the matched cohort, the use of sugammadex significantly reduced the time to extubation compared with neostigmine, with means of 6.0 ± SD 5.3 and 6.6 ± 6.3 min (*p* = 0.0032), respectively (Table 3 and Supplementary Table S2). In addition, the times to leave the operating room (8.5 ± 6.2 vs. 9.0 ± 6.7 min, *p* = 0.0108), arrive in the PACU (18.3 ± 6.0 vs. 18.9 ± 6.6 min, *p* = 0.0088), and the duration of anesthesia (151.6 ± 89.6 vs. 158.5 ± 82.5 min, *p* = 0.0406) were all significantly shorter in the sugammadex group compared with the neostigmine group. There was no notable difference in the length of PACU stay or the duration of surgery between the two groups. 

### 3.3. Adverse Events of the Reversal Agents

The incidence of bradycardia was significantly lower in the sugammadex group (10.2%) compared with the neostigmine group (16.9%; *p* < 0.001) (Table 4). There was no difference in the rates of PONV or oxygen desaturation between the two groups. No patient had continuous positive airway pressure therapy or tracheal reintubation in the PACU. The rates for the use of nasal cannulas and simple masks were similar between the two groups.

### 3.4. Cost-Effectiveness Analysis

The time to extubation costs and personnel costs were significantly lower in the sugammadex group (17.4 ± 15.6 and 25.8 ± 16.3 USD, respectively) compared with the neostigmine group (19.0 ± 18.6 USD, *p* = 0.0032; 27.2 ± 18.7 USD, *p* = 0.0130) (Table 3 and Supplementary Table S1). However, the economic benefits did not outweigh the higher drug costs of sugammadex (186.2 ± 44.7 USD) compared with neostigmine (23.4 ± 7.7 USD, *p* < 0.0001). The total costs in the neostigmine group were significantly lower compared with the sugammadex group, 50.6 ± 21.4 and 212.0 ± 49.5 USD (*p* < 0.0001), respectively.

## 4. Discussion

In this study, we found that the use of sugammadex was associated with a reduced time to extubation compared with neostigmine, although this difference was less than one minute. In addition, patients receiving sugammadex had a shorter time to leave the operating room and duration of anesthesia and a lower incidence rate of bradycardia in the PACU. However, the cost-effectiveness analyses demonstrated that the time-saving benefits of sugammadex could not compensate for its higher price compared with neostigmine. Our results did not support the cost-effectiveness of sugammadex for reversing neuromuscular blockade compared with neostigmine in Taiwan’s healthcare system. 

Several previous studies have examined the economic impact of sugammadex in various clinical settings. A simulation model by Insinga et al. showed that sugammadex could reduce the risk of residual neuromuscular blockade and improve the efficiency of the OR [25]. This benefit might be a result of reduced procedural cancellations, less staff overtime, and increased procedural throughput [28]. In a systemic review of randomized controlled trials, Paton and colleagues demonstrated that sugammadex was only cost-effective if the recovery time was reduced in the OR (assumed value of staff time, £4.44 per minute) [18]. Similarly, Hurford et al. conducted a decision model cost analysis and showed that $8.60 per minute of staff time value was needed to establish the cost-effectiveness of sugammadex in the included studies [12], which was nearly four times the personnel cost in our study. In contrast, Deyhim and colleagues used retrospective data and reported that sugammadex was associated with a shortened recovery time but not meaningful cost-effectiveness, possibly due to the fact that there was no extrapolation to workflow capacity for an increased surgical case volume [20]. The differences in recovery parameters (e.g., time to train-of-four ratio >0.7–0.9, endotracheal extubation, or leaving OR) and staff time value potentially explain the discrepancies in economic analysis results across different studies. 

Previous studies have shown that the cost-effectiveness of sugammadex compared with neostigmine can only be established when two premises are met [6,17,18,19,20,21,22,23]. First, a reduction in recovery time could be achieved by using sugammadex as a routine reversal agent. Second, this time saving could allow for more operating schedules and fewer procedural cancellations. However, it remains difficult to estimate the value of reduced recovery time because little evidence is available to evaluate how the hospital would utilize the saved time and to what extent this would reduce the delay of surgical schedules and overtime pay for the staff.

In our hospital, more than 100 surgical procedures were performed each weekday, and we included both the duration of OR and PACU stay in the cost-effectiveness analyses, as our compact surgical scheduling required both OR and PACU staff to work overtime routinely. Our analyses did not demonstrate the cost-effectiveness of sugammadex, possibly due to the relatively low labor costs of our OR staff. According to open government data, the salaries of consultants and registered nurses in the United States are nearly 3 and 4 times higher than those in Taiwan, respectively [29]. Another possible explanation is the relatively low time saving of sugammadex in our study compared with prior studies [11,12,17,30]. In a retrospective analysis of morbidly obese patients undergoing laparoscopic bariatric surgery, De Robertis et al. demonstrated a reduction in OR occupancy duration by up to 23.3 min when using sugammadex compared with neostigmine [17].

Some previous studies compared the cost of postoperative adverse events between sugammadex and neostigmine, such as unplanned postoperative ICU admission [6], 30-day readmission [10], and PONV [12]. Our study excluded ICU admission from the economic analysis as there were no events in our cohort. The Cochrane review has shown that the use of sugammadex reduces the incidence of PONV compared with neostigmine [1,2]. However, we did not observe such a beneficial effect, similar to some other published data [9,31]. Our analyses demonstrated a lower rate of bradycardia associated with sugammadex, as reported in some previous studies [1,32]. In our study, it was difficult to estimate the potential derived cost of these adverse events due to a lack of accurate data on related management in our electronic medical record system. 

### Strengths and Limitations

Compared with previous studies, our cost-effectiveness analyses were based on a large patient sample using real-world data [6,17,18,19,20]. The data used in the current study were taken from a randomized control trial and have been meticulously validated for analysis. However, there were several study limitations as follows. First, our single-center data warrants external validations to examine the generalizability of the study results, especially in countries that have different labor costs from Taiwan. Second, although propensity score matching has been used to minimize any potential imbalance in baseline patient characteristics between groups, the inherent bias from retrospective observations could not be completely eliminated. Third, we did not evaluate the time to recovery of the train-of-four ratio as there were no detailed records on this in our electronic medical database [12,18]. Some studies reported that postoperative residual neuromuscular blockade was associated with increased rates of ICU admission and pulmonary complications [33,34]. However, we argue that time to extubation can be regarded as a more pragmatic indicator than neuromuscular parameters in daily practice. Fourth, we did not compare the intraoperative consumption of volatile anesthetics between the two groups due to data unavailability. Wu et al. recently showed that the use of rocuronium/sugammadex was associated with lower dosages of volatile anesthetics and opioids compared with cisatracurium/neostigmine [35]. Fifth, a small number of patients (4.1%) in the neostigmine group used cisatracurium in the original cohort. The use of cisatracurium was associated with a shorter time to extubation compared with rocuronium [36], which might bias the unmatched results towards the null. However, this effect should be limited due to its small proportion. Finally, derived costs of adverse events were not evaluated as there was no accurate data available, and the subsequent management of conditions was not standardized.

## 5. Conclusions

We found that the use of sugammadex was associated with shorter operating room turnover times compared with neostigmine, including time to extubation, time to leave the operating room, and duration of anesthesia. In addition, the incidence of bradycardia was significantly lower in the sugammadex group. However, the time saving of sugammadex versus neostigmine was only modest, and the faster patient recovery of sugammadex did not outweigh its higher price. Our real-world data did not support the cost-effectiveness of sugammadex, possibly due to the relatively low labor costs in Taiwan’s healthcare system. Further studies are needed to calculate the derived costs of adverse events related to reversal agents and to build personalized decision-making models based on different patient and surgical risk groups.

## Figures and Tables

**Table 1:** Distributions of baseline patient characteristics of the sugammadex and neostigmine groups.

	Original Cohort					Matched Cohort				
	Neostigmine *n* = 952		Sugammadex *n* = 1635		ASMD	Neostigmine *n* = 892		Sugammadex *n* = 892		ASMD
Age, years	53.9	16.2	56.5	16.1	0.28	53.6	16.0	54.9	16.5	0.08
<20	10	1.1	10	0.6	0.14	4	0.5	9	1.0	0.07
20–39	172	18.1	263	16.1		170	19.1	165	18.5	
40–59	380	39.9	580	35.5		359	40.3	346	38.8	
≥60	390	41.0	782	47.8		359	40.3	372	41.7	
Sex, male	494	51.9	703	43.0	0.18	452	50.7	440	49.3	0.03
Body mass index, kg·m^−2^	25.1	4.7	25.5	4.9	0.09	25.2	4.7	25.2	4.7	<0.01
Body mass index ≥ 30 kg·m^−2^	432	45.4	799	48.9	0.07	411	46.1	413	46.3	<0.01
ASA physical status					0.04					0.06
I	165	17.3	299	18.3		160	17.9	191	21.4	
II	757	79.5	1292	79.0		714	80.0	676	75.8	
III or IV	30	3.2	44	2.7		18	2.0	25	2.8	
Diabetes mellitus	145	15.2	261	16.0	0.02	125	14.0	129	14.5	0.01
Preoperative blood tests										
Creatinine, mg·dL^−1^	1.00	1.24	0.85	0.28	0.51	0.85	0.25	0.90	0.29	0.18
AST, U·L^−1^	26.5	24.2	27.0	30.0	0.34	26.5	24.5	26.4	24.4	<0.01
ALT, U·L^−1^	28.3	28.2	29.6	33.3	0.36	28.4	28.3	29.8	29.7	0.05
Type of surgery					0.17					0.09
Gastrointestinal	162	17.0	330	20.2		155	17.4	183	20.5	
Orthopedic	274	28.8	466	28.5		159	29.0	248	27.8	
Otolaryngological	101	10.6	140	8.6		94	10.5	92	10.3	
Gynecological or urological	194	20.4	288	17.6		177	19.8	165	18.5	
Neurological	76	8.0	187	11.4		74	8.3	78	8.7	
Other	145	15.2	224	13.7		133	14.9	126	14.1	
Type of volatile anesthetics					<0.01					0.01
Sevoflurane	669	70.3	1149	70.3		627	70.3	623	69.8	
Desflurane	292	30.7	503	30.8		274	30.7	278	31.2	
Intravenous anesthetics										
Fentanyl dose, µg	110.8	52.2	113.6	52.9	0.21	112.8	52.3	110.6	50.3	0.04
Rocuronium dose, mg	59.4	29.4	70.1	30.3	0.47	62.6	27.0	62.9	25.1	0.01
Neostigmine, mg	2.6	0.3	0	0	NA	2.6	0.3	0	0	NA
Glycopyrrolate, mg	0.6	0.1	0.1	0.1	0.13	0.6	0.1	0.1	0.1	0.17
Sugammadex, mg	0	0	144.3	36.2	NA	0	0	143.9	36.7	NA

Values were mean ± standard deviation or counts (percent). ALT = alanine aminotransferase; ASA = American Society of Anesthesiologists; ASMD = absolute standardized mean difference; AST = aspartate aminotransferase; NA = not applicable.

**Table 2:** Drug doses and costs for cost-effectiveness analyses.

	Average Dose (Original) (mg)		Average Dose (Matched) (mg)		Cost per Dose (USD·mg−1)
Neuromuscular blocking and reversal agents	N group	S group	N group	S group	
Rocuronium	59.4	70.1	62.6	62.9	0.28
Neostigmine	2.6	0	2.6	0	1.40
Glycopyrrolate	0.6	0.1	0.6	0.1	3.50
Sugammadex	0	144.3	0	143.9	1.17

N group = neostigmine group; S group = sugammadex group. USD = United States dollar.

**Table 3:** Operating room turnover time and cost-effective analyses of the sugammadex and neostigmine groups (matched cohort).

	Neostigmine *n* = 892		Sugammadex *n* = 892		Adjusted Mean Difference ^†^	95% CI	*p*
Operating room turnover time, min							
Time to extubation	6.6	6.3	6.0	5.3	−0.10	−0.17, −0.03	0.0032
Time to leave the OR	9.0	6.7	8.5	6.2	−0.07	−0.13, −0.02	0.0108
Time to arrive at the PACU	18.9	6.6	18.3	6.0	−0.04	−0.06, −0.01	0.0088
Length of PACU stay	48.0	9.3	48.8	13.8	0.02	0, 0.03	0.0785
Duration of surgery	107.0	73.3	101.9	77.6	−0.05	−0.11, 0.01	0.1201
Duration of anesthesia	158.5	82.5	151.6	89.6	−0.05	−0.09, 0	0.0406
Costs, USD							
Personnel ^‡^	27.2	18.7	25.8	16.3	−0.06	−0.11, −0.01	0.0130
Time to extubation	19.0	18.6	17.4	15.6	−0.10	−0.17, −0.03	0.0032
PACU	8.2	1.6	8.4	4.1	0.03	0.01, 0.05	0.0039
Neuromuscular blocking and reversal agents	23.4	7.7	186.2	44.7	2.07	2.05, 2.09	<0.0001
Total ^§^	50.6	21.4	212.0	49.5	1.43	1.40, 1.45	<0.0001

Values were mean ± standard deviation. CI = confidence interval; OR = operating room; PACU = post-anesthesia care unit; USD = United States dollar. ^†^ Adjusted for age, sex, body mass index, and rocuronium dose. ^‡^ Personnel costs = time to extubation costs + PACU costs; ^§^ Total costs = personnel costs + costs of neuromuscular blocking and reversal agents

**Table 4:** Adverse events in the PACU (original cohort).

	Neostigmine *n* = 952		Sugammadex *n* = 1635		*p*
PONV	160	17.7%	307	19.2%	0.36
Bradycardia	161	16.9%	166	10.2%	<0.001
Oxygen desaturation	4	0.4%	9	0.6%	0.78
Use of a nasal cannula	831	87.3%	1428	87.4%	0.94
Use of a simple mask	139	14.6%	255	15.6%	0.50
Use of CPAP	0	0	0	0	NA
Re-intubation	0	0	0	0	NA

Values were counts (percent). CPAP = continuous positive airway pressure; NA = not applicable; PONV = postoperative nausea and vomiting.

## Data Availability

The data presented in this study are available on request from the corresponding author.

## Supplementary Materials

The following are available online at https://www.mdpi.com/article/10.3390/healthcare11020240/s1, Table S1: Personnel costs for cost-effectiveness analyses; Table S2: Operating room turnover time and cost-effective analyses of the sugammadex and neostigmine groups (original cohort).
