# Supplementary material for: Cost-Effectiveness of Sugammadex Versus Neostigmine to Reverse Neuromuscular Blockade in a University Hospital in Taiwan: A Propensity Score-Matched Analysis
Source: Healthcare (Basel). 2023 Jan 12;11(2):240. doi: 10.3390/healthcare11020240 (PMC9859243; doi:10.3390/healthcare11020240)
Supplement: Supplementary file 1 [file healthcare-11-00240-s001.zip › healthcare-2144603-supplementary tables.pdf]

**Table S1**. Personnel costs for cost-effectiveness analyses.

| Staff Members | Monthly Salary (USD) | Monthly Working Time (hour) | Cost per Minute ($USD{\cdot}min^{-1}$) |
|---|---|---|---|
| Surgical resident | 3235.1 | 283 | 0.19 |
| Consultant anesthesiologist | 10783.6 | 160 | 1.12 |
| Circulating nurse | 1617.5 | 160 | 0.17 |
| Scrub nurse | 1617.5 | 160 | 0.17 |
| Nurse anesthetist | 1689.4 | 160 | 0.18 |
| PACU nurse | 1627.3 | 160 | 0.17 |

PACU = post-anesthesia care unit; USD = United States dollar.

**Table S2**. Operating room turnover time and cost-effective analyses of the sugammadex and neostigmine groups (original cohort)

| | Neostigmine *n* = 952 | | Sugammadex *n* = 1635 | | Adjusted Mean Difference† | 95% CI | *p* |
|---|---|---|---|---|---|---|---|
| **Time to extubation, min** | 6.7 | 6.2 | 6.3 | 5.5 | -0.79 | -1.26, -0.32 | 0.0010 |
| **Time to leave the OR, min** | 9.1 | 6.6 | 8.7 | 6.2 | -0.81 | -1.32, -0.30 | 0.0019 |
| **Time to arrive in the PACU, min** | 18.9 | 6.6 | 18.6 | 6.1 | -0.82 | -1.32, -0.32 | 0.0014 |
| **Length of PACU stay, min** | 48.3 | 10.6 | 48.9 | 12.8 | 0.21 | -0.78, 1.20 | 0.6796 |
| **Duration of surgery, min** | 106.3 | 72.8 | 119.9 | 86.4 | -8.28 | -12.75, -3.81 | 0.0003 |
| **Duration of anesthesia, min** | 157.6 | 82.1 | 173.1 | 99.1 | -10.25 | -15.17, -5.33 | <.0001 |
| **Costs, USD** | | | | | | | |
| Personnel‡ | 27.4 | 18.6 | 26.5 | 16.7 | -2.27 | -3.67, -0.87 | 0.0015 |
| Time to extubation | 19.2 | 18.5 | 18.1 | 16.2 | -2.37 | -3.74, -0.99 | 0.0007 |
| PACU | 8.2 | 1.8 | 8.4 | 3.3 | 0.09 | -0.14, 0.33 | 0.4432 |
| Neuromuscular blocking and reversal agents | 22.5 | 8.4 | 188.7 | 45.1 | 162.85 | 160.56, 165.14 | <.0001 |
| Total§ | 49.9 | 21.4 | 215.2 | 50.6 | 160.61 | 157.78, 163.44 | <.0001 |

Values were mean ± standard deviation. CI = confidence interval; OR = operating room; PACU = post-anesthesia care unit; USD = United States dollar.

† Adjusted for age, sex, body mass index, and rocuronium dose.

‡ Personnel costs = time to extubation costs + PACU costs

§ Total costs = personnel costs + costs of neuromuscular blocking and reversal agents
